# The differences in clinical characteristics and natural history between essential tremor and essential tremor plus

**DOI:** 10.1038/s41598-022-11775-8

**Published:** 2022-05-10

**Authors:** Praween Lolekha, Pornpatr Dharmasaroja, Nattaphol Uransilp, Puchit Sukphulloprat, Sombat Muengtaweepongsa, Kongkiat Kulkantrakorn

**Affiliations:** 1 Division of Neurology, Department of Internal Medicine, Faculty of Medicine, Thammasat University, Pathum Thani, 12120 Thailand; 2 Center of Excellence in Stroke, Faculty of Medicine, Thammasat University, Pathum Thani, 12120 Thailand

**Keywords:** Neurological disorders, Movement disorders, Neurodegenerative diseases

## Abstract

The diverse clinical manifestation of essential tremor (ET) has led to the question whether the different phenotypes may affect the clinical outcome and progression. This study aimed to estimate the clinical characteristics and natural history of ET and ET-plus. A total of 221 patients with ET were included, 117 (52.9%) reclassified as ET and 104 (47.1%) as ET-plus. Patients with ET-plus were significantly older in age at onset (*P* < 0.001); had a higher frequency of cranial tremors (*P* < 0.001), neurological comorbidities (*P* < 0.001) and psychiatric comorbidities (*P* = 0.025); more tremor progression (*P* < 0.001); and poorer response to medical treatment (*P* < 0.001) compared to ET patients. Regression analysis revealed that late-onset tremor (OR 11.02, 95% CI 2.79–43.53), neurological comorbidities (OR 3.38, 95% CI 1.56–7.31), psychiatric comorbidities (OR 4.29, 95% CI 1.48–12.44), cranial tremors (OR 2.10, 95% CI 1.02–4.30), and poor response to medical treatment (OR 3.67, 95% CI 1.87–7.19) were associated with ET-plus diagnosis. ET and ET-plus differ in the age of onset, tremor distribution, comorbidities, treatment response rate, and progression. Identifying the ET phenotypes may increase the clinical value in therapeutic strategies and clinical research in the future.

## Introduction

Essential tremor (ET) is a common neurological disorder affecting about 0.3–0.9% of the general population worldwide, and the prevalence increases with advancing age^1,2^. Classically, ET has a benign progression. However, it can be progressive and produce functional disability in some cases. The diagnosis of ET is mainly based on clinical examination, with a clinical characteristic of symmetrical postural and kinetic tremor of the upper limbs with or without head and voice tremors^3^. In the past, ET was considered a monosymptomatic idiopathic neurological disorder. However, emerging evidence indicates that ET is phenotypically heterogeneous with variable motor and non-motor symptoms (NMS).

According to the 2018 consensus statement on tremor disorders by the International Parkinson and Movement Disorder Society (IPMDS), ET has been redefined as an isolated action tremor syndrome of bilateral upper limb action tremor for at least 3 years, with or without a tremor in other locations, and without other neurological signs^4^. Whereas ET patients with additional subtle neurological signs or “soft neurological signs”, such as tremor at rest, impaired tandem gait, questionable dystonic posturing, memory impairment, or other signs that do not suffice to make an additional diagnosis, are considered as ET-plus^4^. These changes aim to provide a better method of differentiating clinical phenotypes of ET and might aid future research studies.

Here we reclassified the diagnosis of ET in Thai patients according to the new classification, emphasizing differences in demographics, clinical manifestations, and progression between ET and ET-plus. This study aimed to estimate the proportion, clinical characteristics, and natural history of ET and ET-plus.

## Materials and methods

A retrospective medical record review for all Thai patients diagnosed with ET (International Classification of Diseases, 10th Revision, Clinical Modification, ICD-10-CM code: G 25.0) at the out-patient department, Thammasat University Hospital, was performed from January 2019 to December 2021. The diagnoses of ET and ET-plus were reclassified according to the consensus statement on the classification of tremors, IPMDS diagnostic criteria^4^. Patients were excluded if they had: (a) inadequate tremor information in their medical records; (b) tremor duration under 3 years; (c) isolated focal voice or head tremors; (d) tremor related to physiological tremor, orthostatic tremor, task- and position-specific tremors, drug-induced tremor, psychogenic tremor; or (e) tremors that related to other neurological diseases such as Parkinson’s disease (PD) or dystonia. Late-onset and senile ET groups were defined as age at tremor onset ≥ 46 years and > 65 years, respectively^5,6^. The reclassification was performed by a movement disorder specialist based on medical records. The protocol for this study was approved by the Human Research Ethics Committee of Thammasat University (MTU-EC-IM-0-151/64). Informed consent was given a formal waive because of the retrospective design by the institutional ethics committee. All the methods were carried out in accordance with the relevant guidelines and regulations.

General demographic data, including sex, current age, age of tremor onset, family history of tremor syndromes, past medical illness, clinical characteristics, and clinical course, were collected. The tremor progression and treatment responsiveness were rated based on the clinical course documented in medical records at 6 months–1 year of follow-up. We redefined tremor progression as static or definite progression and the pharmacological responsiveness as a poor or satisfactory response. The continuous variables were summarized as the mean ± standard deviation (SD) or median, and categorical variables were summarized as numbers and percentages. Comparisons of quantitative data were carried out using the independent t-test or Mann–Whitney test, and qualitative data were compared using the chi-squared test or Fisher’s exact test. The strength of association between nominal variables was determined using the Lambda value. Binary logistic regression analysis was applied to identify significant factors associated with ET-plus. All tests were two-sided, and a *P* value of less than 0.05 was considered statistically significant.

## Results

**Figure 1:**
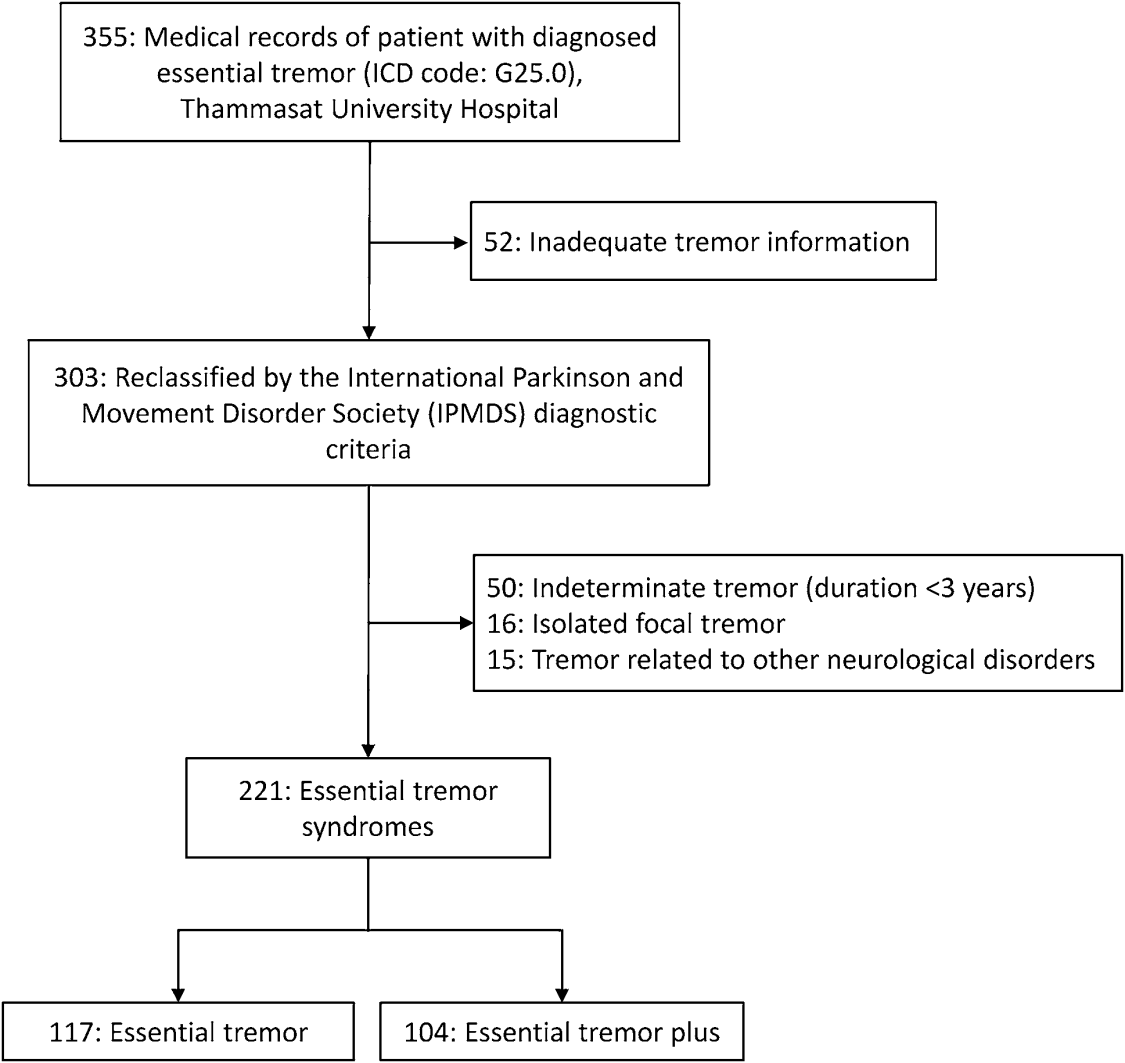
Flowchart of the reclassification of 355 patients diagnosed with ET.

A total of 355 medical records of patients with a diagnosis of ET were reviewed. Of these, 52 (14.6%) records were excluded due to inadequate information on tremors. The remaining 303 (85.4%) were reclassified using the 2018 consensus statement. Eighty-one patients (26.7%) did not meet the diagnostic criteria due to a duration of tremor under 3 years (50), isolated focal tremor (16), and tremor-related to other neurological disorders (15). The remaining 221 (62.3%) patients met the diagnostic criteria of ET syndromes. These included 117 (33.0%) reclassified as ET and 104 (29.3%) as ET-plus (Fig. 1).

In patients who fulfilled the clinical diagnosis of ET syndromes, 167 (75.6%) were diagnosed and followed up by neurologists, and 54 (24.4%) patients were followed up by general practitioners. In contrast, 70.1% of patients who do not meet the diagnostic criteria were diagnosed by general practitioners. There was a strong association between the fulfilled clinical diagnosis and the specialty of the physician who made the diagnosis (lambda value = 0.37).

Regarding topography of tremor manifestations, 209 (94.6%) patients reported relatively symmetrical bilateral hand involvement, 51 (23.1%) had head tremor, 19 (8.6%) had voice tremor, 11 (5.0%) had jaw tremor, 10 (4.5%) had leg tremor, and 10 (4.5%) had both head and voice tremor. In addition, female gender and late-onset ET were significantly associated with cranial tremors compared to male patients (38.2% vs 18.3%, *P* < 0.001) and early-onset ET (33.2% vs 18.2%, *P* = 0.029). In terms of comorbidities, 22 (10.0%) of the patients had a history of stroke, four (1.8%) had epilepsy, 13 (5.9%) had cognitive impairment, and 18 (8.1%) had polyneuropathy. Most ET patients reported no to minimal tremor progression, with only 21 (9.5%) reporting definite progression of their tremor. The response to pharmacological treatments, either beta-blockers or antiepileptics, was approximately 45.7%.

### Comparisons between patients with ET and ET-plus 

In patients who met the diagnostic criteria of ET syndromes, 117 (52.9%) were reclassified as ET and 104 (47.1%) as ET-plus. The mean age of the patients was 67.79 ± 19.03 years, with an age range of 18–96 years. The demographic and clinical characteristics of the patients are shown in Table 1. Patients with ET-plus were significantly older in age (77.06 ± 11.41 vs. 59.55 ± 20.63 years, *P* < 0.001) and age of tremor onset (70.33 ± 11.42 vs. 53.30 ± 20.61 years, *P* < 0.001), and had a higher frequency of cranial tremors (41.3% vs. 19.7%, *P* < 0.001), neurological comorbidities (44.2% vs. 12.0%, *P* < 0.001), and psychiatric comorbidities (21.2% vs. 10.3%, *P* = 0.025) compared to ET patients. ET-plus patients also showed poorer response to pharmacological treatment (76.9% vs. 34.2%, *P* < 0.001) and more frequent progression of their tremor (20.2% vs. 0.0%, *P* < 0.001) compared to ET patients. Regression analysis identified the following factors as significantly associated with ET-plus: late-onset tremor (OR 11.02, 95% CI 2.79–43.53), neurological comorbidities (OR 3.38, 95% CI 1.56–7.31), psychiatric comorbidities (OR 4.29, 95% CI 1.48–12.44), cranial tremors (OR 2.10, 95% CI 1.02–4.30), and poor response to medical treatment (OR 3.67, 95% CI 1.87–7.19) (Table 2). There was no difference in sex, family history of tremor, disease duration, or rate of lower limb tremor between groups.

**Table 1:** Demographic and clinical characteristics of ET and ET-plus patients.

Characteristics	All ET syndrome (n = 221)	ET (n = 117)	ET-plus (n = 104)	*P *value
Sex, male	93 (42.1%)	45 (38.5%)	48 (46.2%)	0.248
**Family history of tremor**				
Recorded (n = 106)	56/106 (52.8%)	30/55 (54.5%)	26/51 (51.0%)	0.713
Not recorded (n = 115)	115 (52.0%)	62 (53.0%)	57 (54.5%)	0.763
Age, years	67.79 ± 19.03	59.55 ± 20.63	77.06 ± 11.41	** < 0.001**
Age of onset, years	61.31 ± 18.91	53.30 ± 20.61	70.33 ± 11.42	** < 0.001**
Late onset tremor (≥ 46)	182 (82.4%)	81 (69.2%)	101 (97.1%)	** < 0.001**
Senile ET (> 65 years)	124 (56.1%)	46 (39.3%)	78 (75.0%)	** < 0.001**
Disease duration, year	6.49 ± 4.89	6.24 ± 5.65	6.77 ± 3.86	0.431
Follow-up by neurologist	167 (75.6%)	80 (68.4%)	87 (83.7%)	**0.008**
Neurological comorbidities	60 (27.1%)	14 (12.0%)	46 (44.2%)	** < 0.001**
Cerebrovascular disease	22 (10.0%)	9 (7.7%)	13 (12.5%)	0.233
Cognitive impairment	13 (5.9%)	0 (0.0%)	13 (12.5%)	< 0.001^a^
Epilepsy	4 (1.8%)	0 (0.0%)	4 (4.0%)	0.048^a^
Polyneuropathy	18 (8.1%)	6 (5.1%)	12 (11.5%)	0.082
Restless leg syndrome	1 (0.5%)	0 (0.0%)	1 (1.0%)	0.471^a^
Psychiatric comorbidities	34 (15.4%)	12 (10.3%)	22 (21.2%)	**0.025**
Anxiety disorders	15 (6.8%)	8 (6.8%)	7 (6.7%)	0.990
Depression	15 (6.8%)	4 (3.4%)	11 (10.6%)	**0.033**
Hallucinations	5 (2.3%)	0 (0.0%)	5 (4.8%)	**0.022**^a^
Cranial tremors	66 (29.9%)	23 (19.7%)	43 (41.3%)	** < 0.001**
Head tremor	51 (23.1%)	18 (15.4%)	33 (31.7%)	**0.004**
Jaw tremor	11 (5.0%)	2 (1.7%)	9 (8.7%)	**0.018**
Voice tremor	19 (8.6%)	9 (8.3%)	10 (9.6%)	0.611
Lower limb tremor	10 (4.5%)	5 (4.3%)	5 (4.8%)	0.849
Static/minor tremor progression	200 (90.5%)	117 (100%)	83 (79.8%)	** < 0.001**
Poor response to treatment	120 (54.3%)	40 (34.2%)	80 (76.9%)	** < 0.001**
Beta-blockers	192 (86.9%)	109 (93.2%)	83 (79.8%)	**0.003**
Antiepileptics	79 (35.7%)	30 (25.6%)	49 (47.1%)	**0.001**
Levodopa	21 (9.5%)	0 (0.0%)	21 (20.2%)	** < 0.001**^a^

*ET* essential tremor.

Significant values are in [bold].

^a^Based-on Fisher's exact test.

**Table 2:** A binary logistic regression model with variables for ET-plus patients.

Variable	B	SE	Wald	df	*P *value	OR	95% CI
Constant	− 3.707	0.726	26.105	1	0.000	0.025	
Late onset tremor	2.400	0.701	11.734	1	0.001	11.024	2.792–43.526
Psychiatric comorbidities	1.457	0.543	7.201	1	0.007	4.293	1.481–12.444
Neurological comorbidities	1.218	0.394	9.559	1	0.002	3.379	1.562–7.311
Cranial tremors	0.742	0.366	4.100	1	0.043	2.099	1.024–4.304
Poor response to treatment	1.301	0.343	14.362	1	0.000	3.671	1.874–7.193

In the ET-plus group, 40 (38.5%) of the 104 patients had multiple soft neurological signs; 47 (45.2%) had mild memory impairment, 43 (41.3%) had rest tremor, 37 (35.6%) had tandem gait impairment, 16 (15.4%) had questionable dystonia, and three (2.9%) had mild dysmetria in the upper limbs. Patients with ET-plus also had a higher frequency of epilepsy, cognitive impairment, depression, and hallucination than patients with ET.

## Discussion

We describe the clinical phenotype and characteristics of 221 patients with ET syndromes. These include 117 (52.9%) reclassified as ET and 104 (47.1%) as ET-plus. Therefore, only one-third of cases initially diagnosed as ET persisted in being diagnosed as ET under the new classification. This observation supported the existence of phenotypic differences in ET. In addition, more than a quarter of patients initially diagnosed with ET did not meet the new diagnostic criteria for ET. These results suggest that, without the defined diagnostic criteria, the concept of ET varies substantially among clinicians. Acknowledgment of the new classification and diagnosis criteria is crucial for improving the value of this syndrome in clinical diagnosis and research.

**Figure 2:**
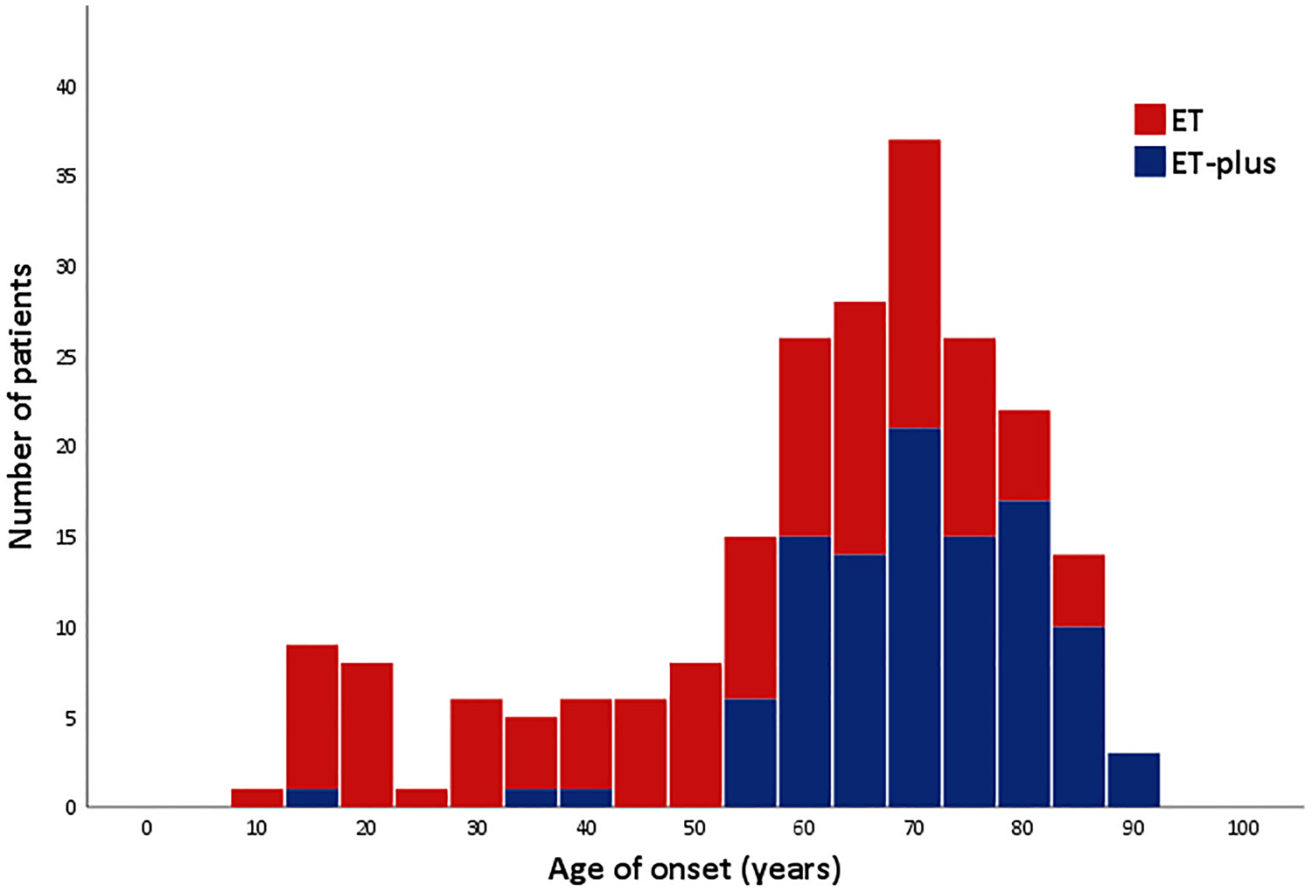
Histogram of the age-at-onset distribution of ET and ET-plus.

To the best of our knowledge, this is the first study to assess the clinical phenotypes, demographics, and characteristics of ET syndromes in Thai patients. Our data showed a concordant prevalence of ET that increased with advancing age^1,2,7^. We found a slightly higher prevalence in females than males, as was previously reported^7^. However, most recent epidemiological studies have found a predominance of affection for the male gender^1,2^. This inverse proportion might be an impact of a higher survived female elderly in the Thai population and conducting a hospital-based prevalence survey. Male patients are less likely to attend a hospital with mild symptoms. We observed a bimodal age distribution for the age of onset with a small early peak in the first and second decades of life and a later rise in the sixth and seventh decades, as described in previous studies^6,8^ (Fig. 2). Late-onset and senile ET were found in over 80% and 55% of the cases, respectively. This distribution might be an effect due to recall bias and the nature of our hospital, a tertiary referral center. A positive family history of ET was found in 50–55% of patients in our study, lower than the previous reports of 60–75%^6,8,9^. This difference might be affected by the amount of missing data from medical records in our study. In terms of the topography of tremor manifestations, our study showed a distribution of tremors concordant with earlier reports^5,8–10^. Head tremor was observed in approximately one-quarter of the patients and predominantly in female patients, while voice tremor was the third most commonly involved body part in ET^5,8,9^.

In this study, we found a slightly higher prevalence of ET compared to ET-plus. Since the diagnosis of ET-plus in our study is mainly based on the presence of soft neurological signs recorded in the medical history, the prevalence of ET-plus in our study might be lower than the true prevalence. However, we could identify several factors associated with ET-plus: older age at onset, the presence of cranial tremors, poor response to medical treatment, and history of psychiatric and neurological comorbidities. These factors agreed with previous studies^11–13^.

ET-plus patients were significantly older in age and age at tremor onset compared to ET patients, resulting in a substantially higher prevalence of comorbidities and degenerative neuropsychiatric disorders. In addition, our ET-plus patients also had a higher prevalence of cranial tremors and a poorer response to medical treatments. These findings were consistent with previous reports that showed that cranial tremors were associated with more severe hand tremors and are less responsive to beta-blockers^9,14^.

An association between ET and dystonia is well recognized but not frequently observed in a clinical setting^8,15^. Only trained neurologists and movement disorder specialists might clinically notice the differences. In a previous cohort database, about half of the ET patients evaluated at the movement disorders clinic had a variety of patterns of associated dystonia, including cervical dystonia, writer’s cramp, spasmodic dysphonia, and cranial dystonia^8^. According to the new consensus statement on tremor disorders, the clinical interpretation of questionable dystonia has been left to investigator^4^. So, the diagnosis of ET-plus is even more challenging with a high rate of discordance^15,16^. The proportion of questionable dystonia in ET-plus patients from the previous studies varied between 0 and 91%, depending on the research methodology and assessment tools^11–13,16–18^. A higher prevalence rate of cranial tremors and poorer responsiveness to medical treatment in ET-plus patients might result from questionable or subtle signs of undetected cranial-cervical dystonia. Regarding tremor progression, all ET patients had no-to-minor tremor progression. In contrast, approximately 20% of ET-plus patients reported worsening tremor with time.

In terms of non-motor features, ET-plus patients had a higher frequency of cognitive and psychiatric symptoms compared to ET patients. Many reports of neuropsychological performance in ET patients have shown a pattern of attentional and executive dysfunction resembling that observed in subcortical dementia, reflecting the disruption in the cerebello-thalamo-frontal network^19^. Alternately, they may be a feature of concomitant neurodegenerative diseases associated with ET-plus, such as PD or Alzheimer’s disease^19^. Regarding psychiatric disorders, depression and hallucination were more common in our ET-plus patients. These mental disturbances may be secondary to more severe tremor symptoms in ET-plus or differences in pathologic brain lesions. While restless leg syndrome (RLS) has been reported associated with ET^20^, our study found only one documented case in the ET-plus group. This result might reflect an unawareness of RLS symptoms in Thai ET patients. Screening of RLS and other NMS with an NMS questionnaire might benefit in early detection and management of NMS in ET patients. A recent study has shown that many components of ET-plus, including motor and non-motor features, correlated with age and tremor duration^21^. This finding indicated that these components could develop in a more advanced stage of ET or even represent a transitional stage between ET and other degenerative diseases. Therefore, ET-plus may represent a disease stage rather than a distinct disease subtype^21^. Interestingly, our patients' disease duration between ET and ET-plus was not significantly different, and all ET patients in our study reported a static in their tremor progression. These findings might suggest that these two phenotypes could represent a difference in a trait condition and rate of progression, or it could be just a pitfall from a recall bias in the retrospective study.

When applying the ET-plus criteria, we faced challenges in a group of patients with signs of both ET and parkinsonism, representing a spectrum of an “ET-PD syndrome”^22^. Most of these patients were excluded from the study because they had a PD diagnosis that might relate to their tremors. The dual-disease pitfall and uncertain definition of soft neurological signs are problematic issues in this classification for clinical implications and epidemiological studies of ET^23,24^. Whether this is a coincidence or a true association, it is possible that longstanding ET patients might develop PD later in life. In a previous comprehensive cohort database of ET patients, approximately 20% of patients with a history of ET later developed parkinsonism^8^. On the other hand, 7.8% of PD patients reported a history of pre-existing ET^25^. To date, the relationship between ET and PD remains controversial^22^. However, there is growing evidence that these two disorders are pathogenically related^26^. A recent study showed a higher prevalence of rapid eye movement (REM) sleep behavior disorder, a prodromal marker of α-synucleinopathies in ET-plus patients, than in the general population^11^. We also observed resting hand and jaw tremors commonly seen in PD in approximately 40% and 9% of ET-plus patients. Moreover, 20% of ET-plus patients required additional treatment with levodopa. These findings suggested that ET-plus might be a prediagnostic phase of PD or a “placeholder” stage before developing other degenerative disorders, reflecting an area of current scientific uncertainty ^11,27,28^.

Pathological and neuroimaging studies found that ET patients had cerebellar atrophy and an increased number of damaged Purkinje cell (PC) axons^10,22^. Furthermore, recent studies have shown disruption of climbing fiber (CF)-PC connections in ET cerebellar cortex, including an abnormal extension of CFs into the outer portion of the cerebellar cortex, increased synaptic contacts of CFs on spiny branchlets of PCs, an increased number of CF-PC lateral crossing fibers^29–31^. These abnormalities are clinical–pathological heterogeneity and associated with tremor severity, sex, and cranial distribution^29–31^. Patients with midline tremors and tandem gait disorders had more cerebellar atrophy and more Purkinje cell axonal swellings with torpedo formation in the cerebellar vermis^32^. Although, a recent postmortem study has not demonstrated pathological differences in cerebellar cortex between ET and ET-plus cases^33^. With additional multiple soft signs, ET-plus patients may have a broader range of pathological lesions in the cerebello-thalamo-cortical circuit. More future research is needed to identify relations between clinical and pathological evidence of ET and ET-plus.

We acknowledge that our present study has some limitations. First, our study was based on data from retrospective chart reviews in one tertiary center, and the number of participants was limited. Many individuals with ET are asymptomatic and are not referred to our center. A prospective, multi-center registry with a larger sample size should be considered in the future. Second, the concept of ET-plus was relatively new and not widely acknowledged by general practitioners and neurologists. Subtle neurological deficits and soft neurological signs such as questionable dystonia and impaired tandem gait may have gone undetected. A video-based assessment with a uniform protocol would provide more detail on neurological signs and improve diagnostic accuracy. Lastly, the patients and clinicians subjectively evaluated the progression and treatment response without using assessment tools. An objective assessment with validated tools and a long-term evaluation is needed for future research protocols.

In summary, ET is a heterogeneous syndrome with variable clinical signs and symptoms. ET and ET-plus patients differ in the age of onset, tremor distribution, comorbidities, treatment response, and progression rate. Identifying the ET phenotypes may increase the clinical value of this syndrome in identifying pathogenetic hypotheses, therapeutic strategies, and clinical research in the future.

## Data Availability

The datasets generated during the current study are available from the corresponding author on reasonable request.
